# An exploratory study of the damage markers NfL, GFAP, and t-Tau, in cerebrospinal fluid and other findings from a patient cohort enriched for suspected autoimmune psychiatric disease

**DOI:** 10.1038/s41398-024-03021-8

**Published:** 2024-07-24

**Authors:** Mikaela Syk, Emma Tornvind, Maike Gallwitz, David Fällmar, Åsa Amandusson, Holger Rothkegel, Torsten Danfors, Måns Thulin, Annica J. Rasmusson, Simon Cervenka, Thomas A. Pollak, Dominique Endres, Ludger Tebartz van Elst, Robert Bodén, Björn M. Nilsson, Gunnel Nordmark, Joachim Burman, Janet L. Cunningham

**Affiliations:** 1 Department of Medical Sciences, Psychiatry, Uppsala University, Uppsala, Sweden; 2 Department of Surgical Sciences, Neuroradiology, Uppsala University, Uppsala, Sweden; 3 Department of Medical Sciences, Clinical Neurophysiology, Uppsala University, Uppsala, Sweden; 4 Department of Surgical Sciences, Radiology, Uppsala University, Uppsala, Sweden; 5 Department of Mathematics, Uppsala University, Uppsala, Sweden; 6 Centre for Psychiatry Research, Department of Clinical Neuroscience, Karolinska Institute & Stockholm Health Care Services, Region Stockholm, Stockholm, Sweden; 7 Department of Psychosis Studies, Institute of Psychiatry, Psychology and Neuroscience, King’s College London, London, UK; 8 Department of Psychiatry and Psychotherapy, Medical Center—University of Freiburg, Faculty of Medicine, University of Freiburg, Freiburg, Germany; 9 Department of Medical Sciences, Rheumatology, Uppsala University, Uppsala, Sweden; 10 Department of Medical Sciences, Neurology, Uppsala University, Uppsala, Sweden

**Keywords:** Molecular neuroscience, Diagnostic markers, Predictive markers

## Abstract

There is growing evidence suggesting that immunological mechanisms play a significant role in the development of psychiatric symptoms in certain patient subgroups. However, the relationship between clinical red flags for suspected autoimmune psychiatric disease and signs of central nervous system (CNS) pathology (e.g., routine cerebrospinal fluid (CSF) alterations, CNS damage markers, neurophysiological or neuroimaging findings) has received limited attention. Here, we aimed to describe the prevalence and distribution of potential CNS pathologies in psychiatric patients in relation to clinical red flags for autoimmune psychiatric disease and psychiatric symptoms. CSF routine findings and CNS damage markers; neurofilament light chain protein (NfL), glial fibrillary acidic protein (GFAP) and total Tau (t-Tau), in CSF from 127 patients with psychiatric disease preselected for suspected immunological involvement were related to recently proposed clinical red flags, psychiatric features, and MRI and EEG findings. Twenty-one percent had abnormal routine CSF findings and 27% had elevated levels of CNS damage markers. Six percent had anti-neuronal antibodies in serum and 2% had these antibodies in the CSF. Sixty-six percent of patients examined with MRI (n = 88) had alterations, mostly atrophy or nonspecific white matter lesions. Twenty-seven percent of patients with EEG recordings (n = 70) had abnormal findings. Elevated NfL levels were associated with comorbid autoimmunity and affective dysregulation symptoms. Elevated t-Tau was associated with catatonia and higher ratings of agitation/hyperactivity. Elevated GFAP was associated with acute onset, atypical presentation, infectious prodrome, tics, depressive/anxiety symptom ratings and overall greater psychiatric symptom burden. In conclusion, preselection based on suspected autoimmune psychiatric disease identifies a population with a high prevalence of CSF alterations suggesting CNS pathology. Future studies should examine the value of these markers in predicting treatment responses.

## Introduction

Comorbid autoimmune disease is overrepresented in patients with psychiatric disorders [1, 2] as are psychiatric symptoms in autoimmune disorders [3]. The risk for psychiatric morbidity is increased many years, possibly decades before the diagnosis of autoimmune disease is established [4]. The emerging field of immunopsychiatry suggests that there are subgroups of patients with psychiatric symptoms that may benefit from treatment targeting underlying autoimmunity [5–7]. Schizophrenia has a strong immunogenetic association [8–10] and immune markers (e.g., interleukin-6) although heterogeneous within the population, have been shown to be associated at the group level with acute psychosis, depression and obsessive-compulsive disorder (OCD) [11–13]. A recent study also described an elevated risk of psychiatric disorders after infection that increased in a dose response manner and with temporal proximity to the last infection [14].

The most striking example of immune-mediated psychiatric symptoms is autoimmune encephalitis. The term denotes several types of central nervous system (CNS) diseases mediated by immune cells and autoantibodies directed against neuronal antigens. Since the discovery of anti-N-methyl-D-aspartate receptor (NMDAR) encephalitis in 2007, a number of anti-neuronal antibodies against both intracellular antigens and cell surface antigens have been described [6, 15, 16]. Factors associated with the development of autoimmune encephalitis include relatively common bacterial and viral infections, and tumors [17, 18].

Patients with autoimmune encephalitis often present with cognitive impairment and psychiatric symptoms [17, 19, 20], and notably, patients with subforms of autoimmune encephalitis may have only psychiatric symptoms [6, 21]. Other immunological CNS diseases may also present with only psychiatric symptoms (e.g., systemic erythematosus lupus, SLE) [22]. However, demonstrations of anti-neuronal antibodies in cerebrospinal fluid (CSF) are relatively rare in broader psychiatric populations [23, 24]. To improve the identification of patients with autoimmune psychiatric disease, several authors have proposed lists of clinical red flags that should lead to further investigation (e.g., with lumbar puncture, neuroimaging) [6, 19]. The term “autoimmune psychosis” and, more recently “autoimmune OCD” have been proposed [6, 7, 25, 26]. In the present study, the term “autoimmune psychiatric disease” refers to all possible known and novel forms of autoimmune processes, including autoimmune encephalitis, with mainly psychiatric manifestations.

Moreover, anti-neuronal antibodies or neuroinflammation can be related to elevated CSF marker levels associated with CNS damage. These CSF markers can indicate neuronal death, axonal loss, or both [27–30]. The CNS damage markers neurofilament light chain protein (NfL), total Tau protein (t-Tau) and glial fibrillary acidic protein (GFAP) are constituent parts of neurons and glial cells that have primarily been used in neurological diagnostics for dementia and demyelinating diseases. High levels of t-Tau and NfL have also been found in patients with autoimmune encephalitis and may predict clinical outcome [31]. The serum NfL level was strongly associated with NMDAR antibodies in patients with psychosis [32]. However, the relationship between clinical red flags for suspected autoimmune psychiatric disease and signs of CNS pathology (e.g., routine CSF alterations, CNS damage markers, neurophysiological or neuroimaging findings) has received limited attention.

This paper aims to describe the prevalence and distribution of potential biological CNS pathologies in a psychiatric patient cohort enriched for suspected immunological involvement. We further explored the associations between CNS damage markers in the CSF, clinical red flags, psychiatric symptoms, neuroimaging and electroencephalogram (EEG) findings.

## Materials, subjects, and methods

### Study design and patient cohort

This is a cross-sectional, observational single-center study (Uppsala University Hospital, Sweden). The reference values for abnormalities are the implemented clinical standards that have been previously established by the respective clinical laboratories. This study was conducted in a clinical setting where abnormal findings were in relation to these standard references, patients with abnormal values were compared to those without abnormal values and an independent control group was not collected. Recruitment was performed in collaboration with the Uppsala Immunopsychiatry Clinic (UIP), which evaluates patients with moderate to severe psychiatric symptoms and clinical signs of suspected immunological involvement [33]. Out of the 167 patients evaluated by the team between 2012 and 2020, 156 (93%) consented to participate in the study. After exclusion criteria (no lumbar puncture (*n* = 17), lack of clinical data (*n* = 1), and patients with post-covid states or mastocytosis that are included in other ongoing studies (*n* = 11)) were applied, 127 patients were included in the present study, shown in Supplementary Fig. 1.

### Psychiatric and neurological assessment

The clinical assessment was performed according to the clinical protocol of the Uppsala Immunopsychiatry Clinic, which has recently been described elsewhere [33]. Briefly it involved a psychiatric interview, a neurological examination, standardized psychiatric symptom assessments and a review of the patient’s medical records. The protocol for retrospective data collection from the medical records is described in the supplementary methods (Supplementary methods). The extent of the assessment varied depending on the patient’s ability to participate and access to medical history. A standard neurological examination was conducted within three months of CSF collection and most often on the same day. Obsessions and compulsions were noted where they caused distress and/or impairment. Psychosis was defined as an expression of hallucinations, delusions or disorganized thought processes or behavior. Cognitive dysfunction was defined as recent-onset memory loss, executive dysfunction or disorientation where the symptoms were observable by the clinician and documented in the medical records. Catatonia was noted for patients reported by the assessing or treating clinician or when the patient had a total Bush-Francis Catatonia Rating Scale (BFCRS) score >3 points. Other psychiatric symptoms, such as agitation, mania, other affective symptoms (depression, anxiety, affect lability, etc.), behavioral regression, psychomotor retardation, tics, sleep dysregulation and anhedonia, were also noted if they had been documented in the clinical assessment. A relapsing-remitting course of illness was defined if the patient history indicated major fluctuations in symptoms and function or had been asymptomatic for more than one month after symptom onset.

The BFCRS and the 24-item Brief Psychiatric Rating Scale-Expanded (BPRS-E) were administered at the time of blood/CSF collection [34, 35]. In 26 patients, the BPRS-E score was estimated retrospectively using medical records at the time of blood/CSF collection. Total score, and a 4-factor model were used for the BFCRS with the following factors: F1: Negative/withdrawal, F2: Automatic, F3: Repetitive/echo and F4: Agitated/resistive [36]. Total score and a 4-factor model were also used for the BPRS-E [37]: F1: Depressed/Anxiety, F2: Psychosis, F3: Negative Symptoms and F4: Activation. Overall psychiatric disease severity was estimated with the Clinical Global Impression (CGI) rating scale in conjunction with the clinical assessment or retrospectively [38]

### Clinical signs of suspected autoimmune psychiatric disease (red flags)

The presence of clinical red flags of suspected autoimmune psychiatric disease was assessed retrospectively, based on previously proposed consensus criteria and red and yellow flag symptoms for possible autoimmune encephalitis/psychosis/OCD [6, 7, 19, 39] (listed in Table 1 and defined in more detail in Supplementary Table 3).

**Table 1:** Patient data (N = 127).

Basic characteristics	Statistics	N^a^
Age in years, median (min; max)	29 (16; 75)	127
Sex, Female: Male (% Male)	65: 62 (49)	127
Family history of psychiatric disorders ^b^, n (%)	75 (59)	127
Family history of autoimmune disorder ^b^, n (%)	37 (29)	127
Clinical red flags		
Atypical presentation, n (%)	95 (75)	127
Rapid onset, n (%)	67 (53)	127
Infectious prodrome, n (%)	49 (39)	127
Comorbid autoimmune disorder, n (%)	43 (34)	127
Motor symptoms, n (%)	18 (17)	107
Any abnormal neurological findings, n (%)	49 (46)	107
New-onset seizures, n (%)	3 (2)	127
Comorbid tumors, n (%)	8 (6)	127
Suspected malignant neuroleptic syndrome, n (%)	2 (2)	127
Catatonia, n (%)	31 (24)	127
Psychiatric presentation		
Psychosis, n (%)	74 (58)	127
Cognitive symptoms, n (%)	74 (58)	127
Obsessions-compulsions, n (%)	53 (42)	127
Tics, n (%)	8 (6)	127
ADHD/ADD	23 (18)	127
Psychomotor retardation, n (%)	20 (16)	127
ASD	19 (15)	127
Intellectual disability	9 (1)	127
Observed affective dysregulation symptoms, n (%)	68 (54)	127
Mania, n (%)	15 (12)	127
Agitation/aggression, n (%)	26 (20)	127
Hypersomnia, n (%)	11 (9)	127
Insomnia, n (%)	45 (35)	127
**CGI**, median (min; max)	5 (1; 7)	120
BPRS-E		
BPRS-E total, median (min; max) ^c^	48 (24; 119)	114
BPRS-E F1: Depressed/Anxiety, median (min; max)	13 (4; 23)	119
BPRS-E F2: Psychosis, median (min; max)	8 (4; 22)	121
BPRS-E F3: Negative Symptoms, median (min; max)	4 (3; 19)	124
BPRS-E F4: Activation, median (min; max)	6 (4; 28)	121
BFCRS		
BFCRS total, median (min; max)	0 (0; 37)	102
BFCRS F1: Negative/withdrawal, median (min; max)	0 (0; 16)	102
BFCRS F2: Automatic, median (min; max)	0 (0; 4)	102
BFCRS F3: Repetitive/echo, median (min; max)	0 (0; 9)	102
BFCRS F4: Agitated/resistive, median (min; max)	0 (0; 6)	102
Serum tests		
S-Anti-neuronal abs, n (%)	7 (6)	122
S-Anti-neuronal abs against surface antigen, n	2 (2)	122
S-Anti-neuronal abs against intracellular antigen, n	5 (4)	122
S-Anti-TPO abs above ref, n (%)	9 (9)	102
Cerebrospinal fluid (CSF) tests		
CSF Anti-neuronal ab, n (%)	3 (2)	125
CSF Anti-neuronal abs against surface antigen, n	3 (2)	
CSF analysis findings, (n %)	26 (21)	127
IgG-indices above ref, n (%)	6 (5)	123
Oligoclonal bands, n (%)	10 ^d^ (9)	116
Albumin quotient (CSF/S) above ref, n (%)	15 (12)	123
Pleocytosis, n (%) ^e^	4 (3)	125
CSF CNS damage markers	33 (27)	124
CSF-NfL above ref, n (%)	14 (11)	124
CSF-GFAP above ref, n (%)	14 (11)	124
CSF- t-Tau above ref, n (%)	16 (13)	120

*AUDIT* alcohol use disorders identification test; *ADHD* attention deficit hyperactivity disorder, *ADD* attention deficit disorder; *ASD* autism spectrum disorder; *DUDIT* Drug Use Disorders Identification Test, *SLE* systemic lupus erythematosus, *BFCRS* Bush Francis Catatonia Rating Scale, *CGI* Clinical Global Impression scale. *Abs* antibodies, *TPO* thyroid peroxidase, *CSF* cerebrospinal fluid, *CNS* central nervous system, *OCBs* oligoclonal bands.

^a^Patients with available data.

^b^1st, 2nd and 3rd-degree relatives, list of included conditions is available in the supplement data.

^**c**^BPRS-E = Brief Psychiatric Rating Scale Expanded (Some patients were not evaluated with all the BPRS-E items. Therefore, some patients lacked a total BPRS-E score).

^d^One of these participants had non-CNS specific OCBs (matching OCBs are present in CSF and serum).

^e^The range of the white blood cell count (WBC) in CSF in the population was 0 to 21 × 10^6^/L. The patients with pleocytosis (n = 4) had the following WBC in CSF: 6, 6, 8, 11 and 21 × 10^6^/L. IgG indices (ref. <0.63). White blood cell count ref < 5 cells per 10^6^/L; Age-related CSF/plasma albumin quotient (ref: age >6 months to 45 years, <6.8; age ≥45 years, <10.2); NfL= Neurofilament light (ref: age <30, <380 ng/L; 30–39, <560 ng/L; age 40–59, <890 ng/L; age 60, <1850 ng/L). GFAP = Glial fibrillary acidic protein (ref: age <20, <175 ng/L; age 20–59, <750; age 60, <1,250); t-Tau = Total Tau (ref: age <18, <250 ng/L; age 18–44, <300 ng/L; age 45+, <400 ng/L).

### Blood and CSF sample collection and analysis

Blood plasma, serum and CSF were collected under non-fasting conditions and stored at -80°C in the Uppsala Biobank. Routine CSF analysis, including IgG indices, age-related CSF/plasma albumin quotient (AQ), oligoclonal bands (OCBs) in serum and CSF and white blood cell (WBC) counts was performed by an accredited medical laboratory (Uppsala University Hospital). Local clinical reference values are established by Uppsala University Hospital, Clinical chemistry and are provided in the footnotes for Table 1. None of the cases showed erythrocyte levels in CSF as an indication of traumatic lumbar puncture. CSF NfL and GFAP concentrations were measured with enzyme-linked immunoassays (ELISAs) as previously described [40, 41], at Sahlgrenska University Hospital. The CSF t-Tau concentration was measured using Lumipulse technology in accordance with the instructions of the manufacturer (Fujirebio, Ghent, Belgium). CSF t-Tau, NfL and GFAP values were compared to local age-dependent reference intervals for CSF provided in the footnotes for Table 1.

Immunofluorescence was used to test for IgG antibodies against NMDAR, leucine-rich glioma-inactivated 1 (LGI1), contactin-associated protein-like 2 (CASPR2), α-amino-3-hydroxy-5-methyl-4-isoxazolepropionic acid receptor (AMPAR) and gamma-aminobutyric acid (GABA) receptors on transfected cells (Euroimmune, Lübeck, Germany). Immunoblotting (line-blot) was performed to test for specific IgG antibodies against Hu, Ri, Yo, Ma2, amphiphysin and CV2. If the immunofluorescence screening indicated the presence of glutamic acid decarboxylase (GAD 65)-antibodies, an ELISA was performed to test for anti-GAD65 antibodies.

### Neuroimaging and EEG

All available brain MRIs were systematically reviewed by a specialist in neuroradiology (DF) blinded to patient data using a dedicated data extraction form. Because the examinations were clinically initiated, the exact protocol used varied between patients, but all the examinations included a FLAIR sequence, a T1-weighted sequence, and diffusion-weighted images. Thirty-nine examinations included T1 images after injection of gadolinium contrast agent. Thirty examinations were performed in 3 T, and the remaining 58 were performed in 1.5 T. One patient was excluded from radiological analysis due to a previously known brain tumor and post-surgical lesions. White matter changes (WMCs) were reported according to localization in periventricular, deep white matter, and juxtacortical areas and graded with the Fazekas scale [42]. Atrophy was graded using the Scheltens’ scale for medial temporal atrophy [43], and the global cortical atrophy scale and enlargement of ventricles secondary to atrophy were noted.

All available standard EEG recordings were systematically reviewed by two specialists in clinical neurophysiology (ÅA, HR), blinded to patient data using a dedicated data extraction form. Three broad categories of EEG abnormalities were documented: (1) background alterations (further subdivided into diffuse slowing, focal slowing, slowing of posterior dominant rhythm, background attenuation, discontinuous EEG, asymmetry and decreased reactivity), (2) periodic and rhythmic patterns (with sub-categorization into generalized and lateralized periodic or rhythmic patterns, respectively) and (3) epileptiform activity (subdivided into generalized, focal, multifocal, electrographic seizures and status epilepticus).

### Statistical analysis

The Mann-Whitney test was used to compare continuous or ordinal variables between patient subgroups. The Chi-square test (or Fisher’s test when appropriate) was used for categorical variables. Statistical significance was defined as p < 0.05. Because of the exploratory approach of the statistical analyses, no correction for multiple testing was performed. NfL, GFAP, t-Tau and AQ were binary, categorical variables that were evaluated against age-specific reference values therefore, no further correction was performed for age. Statistical analyses were performed using IBM SPSS Statistics software (SPSS v27, IBM) and R 4.0.3 (R Core Team, 2020). To assess the predictive performance of clinical factors, random forest models [44] were fitted with binary variables indicating the presence or absence of elevated levels of each of the three CNS damage markers as response variables. The predictive importance of the explanatory variables was assessed using the out-of-bag error rate.

### Ethical declaration

The research was conducted in accordance with the World Medical Association Declaration of Helsinki. Ethical approval was acquired by the Regional Ethical Review Board in Uppsala, Sweden; Dnr 2012/081 and 2014/148. Written informed consent was obtained from all participants.

## Results

### Description of the patient cohort

Overall, patients had moderate-severe psychiatric symptoms at assessment. The available clinical and assessment data as well as serum and CSF findings are presented in Table 1. Neurological examinations were performed for 107/127 (84%) patients. Overall, 44% of the patients had documented abnormal findings in the neurological exam. Specifically, 17% had abnormal motor findings (e.g., dyskinesia, tremor, dystonia), 14% had balance/coordination abnormalities, 12% had sensory findings (e.g., paresthesia, hypoalgesia/ hyperalgesia), 11% had reflex abnormalities, 9% had eye movement abnormalities, and 6% had motor weakness. In 122 of 127 (96%) patients, one or more clinical red flags were identified; 14%, 59%, and 22% had one, two, or three or more red flags, respectively. In 21% of the patients, routine CSF abnormalities were detected; 14/127 (11%) had CSF signs of neuroinflammation (increased white blood cell counts/IgG indices or CSF-specific OCBs). Overall, 27% had one or more elevated CNS damage marker(s) in the CSF (i.e., NfL, GFAP or t-Tau). Anti-neuronal IgG antibodies were detected in serum or CSF in 8/127 (6%) of patients and these results are described in Supplementary Table 5 and Supplementary Table 6. For the distribution of the CSF variables in the total population, see Supplementary Table 7. Supplementary Table 8 provides an overview of the psychiatric symptomatology at the individual level.

### Neuroimaging and EEG

**Fig. 1:**
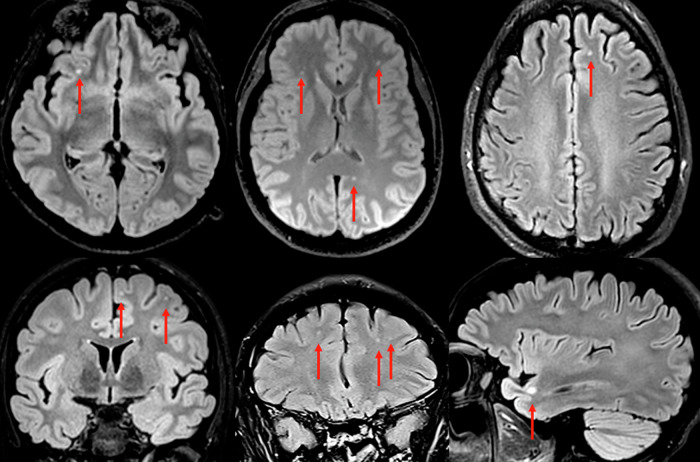
FLAIR images from brain MRI in six representative cases. All six cases have non-specific but distinct and well-demarcated spherical white matter changes. This was a common finding in the study, and more than expected for the age group. The most prevalent locations were deep white matter in frontal and parietal regions. The bottom right image shows a juxtacortical lesion in the temporal lobe. Juxtacortical lesions are rarely caused by small vessel disease, but rather associated with inflammation.

In 58 of the 88 MRIs (66%) alterations were detected; 41% had any form of white matter changes (WMCs), 15% were in periventricular areas, and 40% were in deep white matter, of whom 10 had at least one juxtacortical lesion. The most common type of WMCs was punctate or rounded hyperintensities in the deep white matter, with dominance for the frontal lobes. Sample images from six representative cases are shown in Fig. 1. In addition, 50% of the patients showed some form of atrophy; 31% had at least grade 1 medial temporal lobe atrophy and 41% had at least grade 1 global cortical atrophy. Unmatched oligoclonal bands (OCBs) were more common in patients with WMCs (24% vs. 2%, p = 0.002). No patient had increased cortical signal intensity in the medial temporal lobes. Pathological contrast enhancement combined with unclear parenchymal lesions was observed in 2/88 (2%) cases.

Among the large group of patients (n = 67) who were ≤40 years at the magnetic resonance imaging (MRI) examination, 33% had some form of WMCs and 42% had at least grade 1 atrophy. Among patients with WMCs, six were younger than 25 years at the time of MRI. Of these individuals, five had deep WMCs, and two had at least one juxtacortical component. For the distribution of MRI white matter changes (WMCs) in relation to age categories, see Supplementary Table 2.

EEG data were available for 70 cases. Overall, 27% had abnormal EEG findings. Background abnormalities were the most common findings and were categorized as follows: diffuse slowing (n = 12), focal slowing (n = 9), slow posterior dominant rhythm (n = 1), background attenuation (n = 1) and asymmetry (n = 1). Generalized rhythmic patterns were observed in 4%. Epileptiform discharges were seen in two cases, where one was generalized and the other focal. Six patients (9%) were previously diagnosed with epilepsy, two of whom had pathological EEG findings. One of these cases had background abnormalities and the other had background abnormalities and epileptiform discharges. Of the 70 patients with EEG recordings, 36 (51%) had documented ongoing treatment with psychotropic drugs (e.g., antipsychotics, benzodiazepines or nonbenzodiazepine hypnotics); 11/19 (58%) of patients with pathological EEG findings had documented treatment with psychotropic drugs, primarily antipsychotics and another two had been treated with anti-epileptics.

### CNS damage markers in CSF in relation to clinical red flags for autoimmune psychiatric disease, psychiatric manifestations, neuroimaging and EEG findings

The clinical variables were related to different CNS damage markers, see Supplementary Table 1A–D for a complete overview of the relationships. In summary, elevated NfL levels were more common in patients with comorbid autoimmune disorders than in those without (21% vs. 6%, p = 0.018). In contrast, elevated NfL levels were less common in patients with infectious prodrome (4% vs. 16%, p = 0.046). NfL was associated with observable symptoms associated with affect dysregulation (19% vs 3%, p = 0.008) but was less prevalent in patients presenting with OCD (4% vs 17%, p = 0.022). Elevated t-Tau was more common in the patients with catatonia (27% vs. 9%, p = 0.026). These cases also had higher scores on the BPRS-E F4: Activation (p = 0.033) and BFCRS F4: Agitated/resistive (p = 0.041). All cases with elevated GFAP levels had an atypical disease presentation (15% vs. 0%, p = 0.021), and the majority had an infectious prodrome (21% vs. 6%, p = 0.008) or rapid onset of psychiatric symptoms (17% vs. 5%, p = 0.044). Elevated GFAP levels were related to higher total BPRS-E score (p = 0.033) and these patients were more likely to suffer from tics (50% vs 9%, p = 0.006).

Intellectual disability was present for 9 cases (5 classified as mild and 4 as moderate). Elevated NfL levels were present in 3/9 cases (2/3 were classified as mild) and were greater than expected (p = 0.03). The diagnoses of attention deficit hyperactivity disorder (ADHD), attention deficit disorder (ADD) or autism spectrum disorder (ASD) were not related to the respective damage markers. There were no significant associations between reported family history and elevated damage markers; however, there was a trend toward a negative association between elevated NfL levels and a family history of psychiatric disorders (p = 0.052). There was a wide range of symptom duration with 42% reporting symptoms already in childhood and 25% with symptom onset or worsening within the past year. Symptom duration did not predict elevated CNS damage markers.

No general associations were detected between the presence of MRI findings and CNS damage markers. EEG background abnormalities were more common in patients with elevated NfL levels (60%) than in patients with normal NfL levels (23%, p = 0.025).

### Machine learning models

**Fig. 2:**
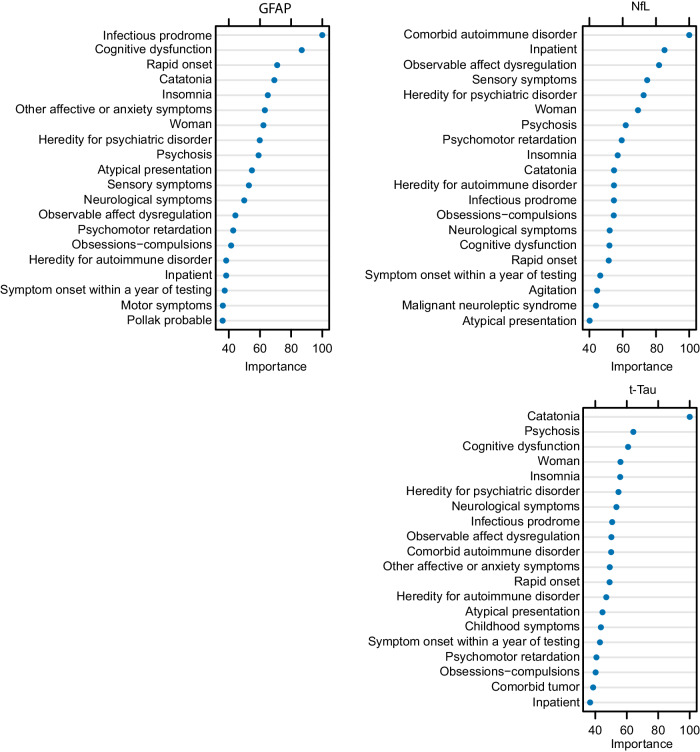
The relative predictive importance ranking of clinical factors in random forest models fitted with binary variables indicating the presence or non-presence of elevated levels of each of the three CNS damage markers as response variables. A rank of 100 is the most important, and lower numbers showing the relative importance of different variables compared to the most important predictor. Where variables used in all models vs not used in the models at all are ranked 100 respective 0.

**Fig. 3:**
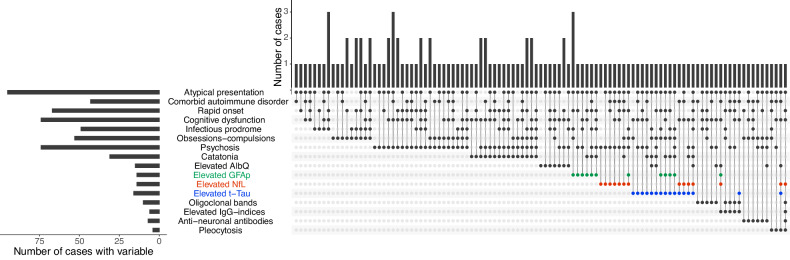
Upset plot of distribution of categorical variables in patients where the top row shows the number of cases with each distinct combination of symptoms/red flags/biological marker. The plot illustrates the low degree of overlap of the CSF findings with each other which may indicate different underlying biology and high overlap of the psychiatric symptom groups; OCD, psychosis and catatonia in this enriched population.

The clinical dilemma is to select patients who should be further investigated with CSF analyses. The clinical variables and red flags were included in random forest models and by repeatedly testing random subsets we identified and ranked the variables in order of importance. A score of 100 was the most important variable, and lower numbers indicate the relative importance of different variables compared to the most important predictor. The variables with the highest importance were Comorbid autoimmune disorder, Infectious prodrome, Catatonia symptoms for NfL, GFAP and t-Tau, respectively (Fig. 2). As a measure of effect size, we identified the clinical features with the highest predictive value for elevated levels of each CNS damage marker by determining the change in predictive accuracy when the variable was removed from the models (Supplementary Fig. 2). For NfL levels, psychomotor symptoms, psychosis and observed affective dysregulation were the most predictive factors. For t-Tau, catatonia symptoms were the most positive predictive factor while psychomotor retardation was a negative predictor (and agitation emerged as a strong predictive factor when psychomotor retardation was excluded from the models). For GFAP, the most important predictive variables were infectious prodrome and atypical presentation. The absolute contribution of each factor to the models relative to the frequency of the variable in the population is shown in Fig. 2 and Supplementary Fig. 2. An UpSet plot showing the distribution of variables in these cases is shown in Fig. 3.

### Autoimmune and infectious diagnoses

The investigations led to a diagnosis of definite NMDAR encephalitis (n = 3), neuropsychiatric SLE (n = 3), multiple sclerosis (n = 1), neuroborreliosis (n = 2), frontotemporal dementia (n = 1) and Wernicke-Korsakoff syndrome (n = 1). The presence of multiple and shifting psychiatric features, and psychiatric diagnosis-based groupings did not predict the number of red flags (see Supplementary Table 8). Notably, three cases with NMDAR encephalitis presented with psychosis, OCD and affective dysregulation, respectively and two of these patients met the Graus criteria for definite NMDAR encephalitis [39]. The patient who did not meet the criteria did not have positive focal neurology, MRI or EEG findings consistent with encephalitis.

**Fig. 4:**
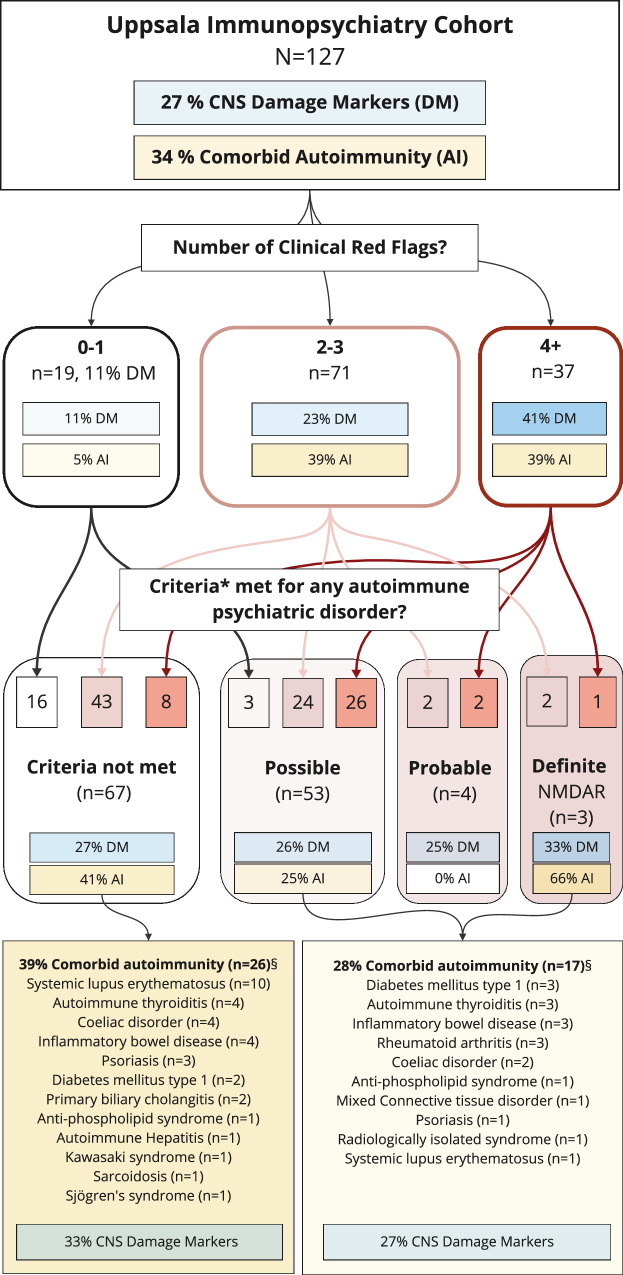
Overview of the patient cohort in terms of CNS damage markers, autoimmunity, red flags and psychiatric auotimmune disorder. TThe flow-chart shows the distribution of patients relative to number of red flags vs. grouping according to combined suggested criteria for autoimmune psychiatric disorder, comorbid autoimmune disorders (AI) and elevated CNS damage markers (DM).

The cases were individually assessed for the fulfillment of the previously suggested diagnostic criteria for autoimmune psychosis, OCD or other psychiatry by applying the same criteria for the latter to other psychiatric disorders [6, 7] (Supplementary Table 4). Combining these groups, 47% met the criteria for a possible autoimmune psychiatric disorder. A large proportion of patients with comorbid autoimmune disorders, including ten of the eleven patients with SLE, did not meet these criteria. This is despite the fact that all cases met 2 or more of the 19 definitions for neuropsychiatric manifestations of SLE based on the American College of Rheumatology [45] and 7 cases met the more restrictive criteria that include only seizures and psychosis [46] (see Fig. 4).

## Discussion

An emerging spectrum of immunological mechanisms is relevant for clinical psychiatry. Clinicians need tools to select patients for further investigations and to improve the understanding of the relevance and implications of abnormal CSF findings. Here, we aimed to describe the prevalence and distribution of potential central nervous system (CNS) tissue damage and other pathologies in a patient group with severe and complex illness and limited effects of standard treatment. This observational study revealed that more than a quarter of patients with clinical signs of suspected autoimmune psychiatric disease had elevated biomarkers of CNS tissue damage that could not be explained by age or comorbid neurodegenerative disorders. The potential differential diagnostic value is exemplified by the associations between elevated NfL levels and underlying autoimmune disorders, t-Tau and catatonia with features of excitation, and GFAP and prodromal infections. Additionally, 21% of the patients had CSF signs of neuroinflammation and/or BBB dysfunction. Exploratory analyses suggested that different clinical features were related to individual damage markers in the CSF. Using standard clinical testing, anti-neuronal antibodies against established antigens were detected in the CSF in 2% of the cases. Moreover, abnormal neuroimaging findings were strikingly frequent, especially in younger patients. Taken together, the overall findings suggest underlying biological groups that extend beyond several traditional psychiatric diagnostic boundaries and may be further developed into tools to inform future diagnostics and understanding of the distinct underlying mechanisms involved.

11% of the patient population had elevated NfL levels, and 13% had increased t-Tau levels in the CSF. Both of which are major cytoskeletal constituents of neuronal cells and axons [47, 48], the release of NfL or t-Tau into the CSF can indicate axonal or neuronal injury [47]. Patients with a known comorbid autoimmune disorder at the time of evaluation (e.g., SLE, IBD) were more likely to have elevated NfL levels in the CSF, which is congruent with being a biomarker for cerebral involvement in patients with SLE [49, 50]. These findings are congruent with the suggestion that elevated NfL levels in the CSF should also lead to suspicion of autoimmunity against targets in the CNS. Notably, the peripheral manifestations of the known comorbid autoimmune disorders were considered well-treated at the time of assessment in this study. Elevated NfL levels were also positively associated with affective dysregulation. Catatonia, especially in the presence of agitation and resistance was associated with elevated t-Tau levels. These findings warrant further investigation of the potential role of NfL and t-Tau levels and neuronal/axonal damage and disinhibition in the pathophysiology of affective dysregulation and catatonia, especially in cases with features of excitation.

One in ten, predominantly younger patients, had elevated levels of GFAP in the CSF. GFAP is the main intermediate filament protein in mature astrocytes. It has a structural role in the cytoskeleton and various other important functions of the cell, including an essential role in maintaining an intact BBB [27] and is a marker for traumatic brain injury [51], early neurodegenerative disorders [52], multiple sclerosis and other CNS disorders [53]. In response to CNS pathology, astrocytes react with astrogliosis and increased expression of GFAP [27, 54]. The observed link between infectious prodrome and elevated levels of GFAP in CSF could theoretically be the result of astrocyte activation, astrogliosis or glial loss [55]. Elevated GFAP levels were positively associated with a greater psychiatric symptom burden, specifically anxiety/depression scores, a finding that is congruent with previous reports of elevated GFAP levels in CSF in patients with major depression [56]. Prodromal infection is not one of the previously suggested criteria for possible autoimmune encephalitis or autoimmune psychosis [6, 39] but it is included in the proposed criteria for possible autoimmune OCD [7]. These criteria were formulated in a series of meetings of international experts in the field based on clinical experience in internal discussions in the context of a non-formal kind of Delphi procedure with iterative criteria formulations and internal assessments and available published data. These criteria are preliminary and the intention is to further refine as data accumulates. Infections are potential triggers of autoimmune diseases and encephalitis and previous studies have reported links between anti-NMDAR encephalitis and viral infections [15, 57]. Interestingly, elevated GFAP levels were also associated with increased levels of anxiety and the occurrence of tics, which are symptom criteria for Paediatric Acute-onset Neuropsychiatric Syndrome (PANS). To date, only one study has investigated GFAP levels in a small cohort of children diagnosed with PANS, but only in the serum, with negative findings [58]. The diagnostic and prognostic implications of elevated GFAP in CSF as well as the underlying mechanisms and role in pathogenesis are still unclear, but given the links to other CNS disorders, further study is needed.

It has become increasingly apparent that many cases with autoimmune encephalitis, especially those with psychiatric presentations, do not exhibit specific radiological findings [6, 21]. For example, no cases in this study had medial temporal lobe hyperintensities. The most prominent radiological finding in this study was a high prevalence of WMCs, mostly small rounded foci in the frontal deep white matter of young patients. When discrete, this finding is usually dismissed as non-specific and can be either ignored or even omitted from the radiological report. It is often interpreted as chronic ischemia in elderly patients, and migraine is often a suspected cause in younger patients. However, it is also a known feature of SLE and a wide range of other neuroinflammatory diseases [59]. Ten patients had juxtacortical changes, usually associated with inflammation rather than chronic ischemia or other forms of degeneration. The prevalence of WMCs was more than three times greater than the reported prevalence in a recent meta-analysis of patients with first-episode psychosis [60]. Due to the high prevalence of WMCs and atrophy in this study, even among the youngest patients for whom chronic ischemia is less plausible, we hypothesize that the imaging findings reflect possibly relevant pathophysiology in at least some of these patients.

EEG background abnormalities were the most common pathological EEG findings in patients with EEG recordings in our cohort. EEG background abnormalities were also more common in patients with elevated NfL levels than in patients with normal NfL levels. Slow background activity is one of the most common EEG findings in patients with psychiatric disorders and is often considered secondary to ongoing pharmacological treatment [61]. However, psychotropic drugs were not significantly more common in the group of patients with EEG abnormalities compared to all patients with EEGs. Moreover, slow background activity can also be observed in a wide range of pathological conditions, including encephalitis and neurodegenerative disorders and may be linked to blood-brain barrier dysfunction [62, 63]. While EEG background abnormalities are isolated findings that must be interpreted with caution, in the context of other pathological findings, they may provide support for organic brain disease.

The random forest models indicated that several red flags are informative helping to select patients for further investigation as they predict elevated CNS damage markers. The results also indicate underlying biological heterogeneity in patients with CSF alterations as different red flags emerge for the respective CNS damage markers. Thus, the notion of red flags should continue to be a work in progress. In this respect, the criteria are intended to identify patients with an indication for further evaluation rather than as a diagnostic criterion alone. Therefore, it may be argued that sensitivity should be prioritized over specificity.

The findings of the anti-neuronal antibody and CSF pathology tests presented here are in line with previous findings indicating that known anti-neuronal antibodies in CSF are rare in psychiatric populations, even when they are enriched for clinical red flags [23, 24, 64–66]. Nevertheless, because anti-neuronal antibodies were measured using a fixed cell-based assay, which has a lower sensitivity than live cell-based assays [67], the prevalence of these antibodies may have been underestimated in this study [67, 68]. Importantly, currently available tests were developed for anti-neuronal antibodies with high relevance for neurological presentations. As shown in previous case studies, psychiatric disease-related antibodies are likely to be discovered with new methods [23, 69].

Although only two cases met the Graus criteria for encephalitis [39], many of the other cases had at least some findings suggesting possible autoimmune mechanisms and available preliminary guidelines for autoimmune psychosis [6] or OCD [7] do not identify many of the cases we suspect are related to autoimmune mechanisms. For example, it is well established that psychiatric symptoms often occur secondary to SLE [2, 70, 71] but only one of the eleven patients with comorbid SLE in this cohort met the criteria for even a possible autoimmune psychiatric disorder. All patients with SLE had two or more of the 19 neuropsychiatric manifestations in the 1999 classification for neuropsychiatric SLE by the American College of Rheumatology [45]. The symptoms were most often catagorised as diffuse, and neuroimaging and/or CSF studies were inconclusive. The distinction between psychological reactions and SLE-related pathophysiological processes was based on judgment of the multidiciplinary team and recommendations from The European Alliance of Associations for Rheumatology [72] and was strongly based on the clinical evaluation of the symptoms and trajectory. A comparative population of patients with SLE without CNS symptoms is needed to confirm the potential high value of CNS damage markers in this context. A recent meta-analysis showed that even other forms of autoimmunity may manifest as psychiatric symptoms that respond to treatment targeting the immune system [5] and suggested that other immunological mechanisms, such as the direct influence of cytokines and chemokines or autoantibodies on brain function as well as several secondary mechanisms, may contribute to the psychiatric phenotypes. Interestingly there was a dose-related relationship between the number of red flags and the presence of elevated CNS damage markers, suggesting clinical relevance. The combination of clinical red flags, CNS damage markers and other evaluations should be considered to optimize the clinical value of these markers, which must ultimately be determined in clinical treatment trials.

### Limitations and strengths

Patients were examined at different time points from symptom onset and may represent different stages of the disease process, which may influence the prevalence of abnormal CSF findings. Complete psychiatric and neurological examinations were not always available. Comorbid autoimmune disorders were based on confirmed diagnoses at the time of data extraction and did not include patients with only non-specific symptoms or laboratory signs of autoimmunity. Longitudinal observations of the study participants may reveal the presence of additional systemic autoimmune disorders. The retrospective data introduced the risk of recall bias. The categorization of clinical red flags was based on specified recommendations from previous studies. However, implementation at the individual level was more difficult than initially expected for more subjective categories (e.g., atypical presentation), which may negatively affect the reproducibility of the results. Patients were generally referred to the clinic because of unsatisfactory treatment responses but we did not further operationalize the criterion “treatment resistance despite guideline-based therapy”. This is a general practical problem in defining and establishing treatment resistance because of the different definitions available for different phenotypes in suspected secondary cases who do not fit well into the classical phenotypic categories. MRI and EEG data were missing, which may have reduced the power to detect associations and introduced selection bias in these analyses. The prevalence of abnormal CSF damage markers did not significantly differ between the groups with/or without MRI/EEG, with the exception of cases with elevated GFAP, for whom all cases have had MRI examinations—this selection bias is likely due to the high frequency of sudden onset of symptoms in this group. The laboratory findings are categorized according to the standard age-corrected clinical reference values. A matched population with severe psychiatric disease but without red flags for immunological involvement was not available for comparison. However, the descriptive statistics and models show however that these markers discriminated within the patient cohort clusters of patients with other clinically relevant variables providing internal validity.

The strengths of this study include CSF analyses in a large enriched cohort, the use of a multimodal approach, the use of structured instruments for symptom evaluation and retrospective data collection. The high variability within the cohort allows us to compare and contrast the respective clinical features. The inclusion of severely ill patients, an underrepresented group in clinical studies, increases the clinical relevance of the results. Selecting patients who should undergo further investigation with CSF analysis is a clinical challenge. The study provides clear indications that clinical features such as catatonia, infectious prodrome and comorbid autoimmunity are linked to CSF findings and provides additional support to motivate these investigations.

## Conclusions

Our data indicate that elevated levels of CNS damage markers are frequent in patients with suspected autoimmune psychiatric disease and are differentially associated with autoimmunity, infectious prodrome, catatonia features and other psychiatric symptoms. We, therefore, suggest that these markers be included in future studies aimed at the biological stratification of patients and further evaluated for their diagnostic value for identifying treatable autoimmune or neuroinflammatory conditions with psychiatric manifestations.

### Supplementary information

Supplementary methods: Protocol for retrospective data collection from medical records, CSF routine analysis reference values, and CNS damage biomarkers reference values

Supplementary Tables 1–7 and Supplementary Figure legends

Supplementary Figure 1. Flow-chart of the inclusion process

Supplementary Figure 2.Effect size for the predictive importance of the explanatory variables

Supplementary Table 8: Overview of psychiatric diagnosis and clinical variables in relation to CSF damage markers

## Data Availability

Due to institutional restrictions, the data of this project cannot be shared on a public repository. Instead, the data can be made available upon request on a case-by-case basis as allowed by the legislation and ethical permits. Requests for access can be made to the corresponding author.

## Code availability

The code used to create the models is available upon request.
