# Supplementary material for: An exploratory study of the damage markers NfL, GFAP, and t-Tau, in cerebrospinal fluid and other findings from a patient cohort enriched for suspected autoimmune psychiatric disease
Source: Transl Psychiatry. 2024 Jul 24;14:304. doi: 10.1038/s41398-024-03021-8 (PMC11269634; doi:10.1038/s41398-024-03021-8)
Supplement: Supplementary file 1 — Supplementary methods: Protocol for retrospective data collection from medical records, CSF routine analysis reference values, and CNS damage biomarkers reference values [file 41398_2024_3021_MOESM1_ESM.docx]

### **Supplementary Methods - Syk and Tornvind et al**

| **Protocol for retrospective data collection from medical records** | | | |
| --- | --- | --- | --- |
| 1. **Identification and dates** | | | |
|  | **Explanation/**  **examples** | **Data** | **Comments/**  **specification (text)** |
| **ID number** |  |  |  |
| **Data collection date** |  |  |  |
| **Name of the data collector** |  | - |  |
| **Baseline date** | Date of lumbar puncture. If not available, date of blood sample collection. |  |  |
| **Date of symptom onset** | Retrospective estimation |  |  |
|  | | | |
| 1. **Symptoms** | | | |
|  | **Explanation/examples** | **Data** | **Comments/**  **specification (text)** |
| **Clinical psychiatric symptoms** | Based on documentation of mental status and anamnesis in the medical records | Yes=1  No=0 |  |
| **Psychotic symptoms** | Hallucinations, delusions and/or disorganized behavior or thought/speech  Should be clearly described as psychotic symptoms by the assessing personnel |  |  |
| **Catatonic symptoms** | Should be clearly described as catatonic symptoms by the assessing personnel |  |  |
| **Obsessions/**  **compulsions** | Should be clinically significant (e.g., loss of function) |  |  |
| **Cognitive dysfunction** | Recent-onset memory loss, executive dysfunction or disorientation with negative impact on function. If yes, specify |  |  |
| **Observable emotional dysregulation** | Affective symptoms observable in mental status (e.g., affect lability, clinically relevant shifts in mood unrelated to situation, episodes of hysteria), If yes, specify |  |  |
| **Psychomotor retardation** | Should be clearly described by the assessing personnel |  |  |
| **Agitation and/or aggression** | Should be clearly described by the assessing personnel |  |  |
| **Mania** | Should be clearly described by the assessing personnel |  |  |
| **Anhedonia (severe)** | Severe loss of initiative or passivity |  |  |
| **Hypersomnia** | Should be clearly described by the assessing personnel. |  |  |
| **Insomnia** | Should be clearly described by the assessing personnel. |  |  |
| **Tics** | Should be clearly described by the assessing personnel, |  |  |
| **Other significant psychiatric symptom or abnormal behavior** | E.g., self-harm or suicidal behavior |  |  |
| **Main symptoms** | A brief summary of the main symptoms | - |  |
| **Clinical neurological symptoms** | Documented in neurological examination or anamnesis. Specify if observed in neurological examination. | Yes= 1  No= 0 |  |
| **Abnormal finding in neurological examination** | If yes, specify |  |  |
| **Abnormal eye movements** | E.g., nystagmus, incomplete eye movements |  |  |
| **Paresis or paralysis** | Reduction in muscle strength or complete paralysis, If yes, specify |  |  |
| **Speech impairment** | E.g., aphasia |  |  |
| **Motor symptoms** | E.g., rigidity, tremor, dystonia, dyskinesia, tics, stereotypical movements, myoclonus, hypokinetic movement, increased tonus, trismus. If yes, specify |  |  |
| **Sensory symptoms** | E.g., decreased or increased sensibility, allodynia |  |  |
| **Balance or coordination impairment** | E.g., dysdiadokokinesia, difficulties with balance, ataxia |  |  |
| **Positive Babinski’s test or abnormal reflexes** | E.g., positive Babinski, general hyperreflexia or clonus |  |  |
|  | | | |
| 1. **Course of illness** | | | |
|  | **Explanation / examples** | **Data**  Yes=1  No=0 | **Comments/**  **specification** (text) |
| **Acute onset** | Onset or escalation of new and severe psychiatric symptoms within three months. E.g., described as “acute”, “abrupt” or “sudden onset” in the medical records. Add specification concerning time-period. |  |  |
| **Atypical presentation** | E.g., atypical age, atypical progress of symptoms, atypical presentation of symptoms (does not agree with the progress or presentation of known psychiatric disorders). |  |  |
| **Prodromal infection or symptoms of inflammation/**  **autoimmunity** | Specify when and what in text |  |  |
| **Relapsing-remitting course of illness** | If the patient history indicates major fluctuations of symptoms and function or if the patient has been asymptomatic for more than one month after symptoms onset |  |  |
| **Persistent course of illness** |  |  |  |
| **Mixed course of illness** | E.g., some symptoms remain constant but there are fluctuations in severity OR some symptoms are constant but others come and go in episodes |  |  |
|  | | | |
| 1. **Descriptives** | | | |
|  | **Explanation / examples** | **Data**  Yes=1  No=0  F=1  M=0 | **Comments/**  **specification** (text) |
| **Age** | At baseline, in years |  |  |
| **Sex** | Male (M)/Female (F) |  |  |
| **Psychiatric comorbidity** | Diagnoses in the medical records (e.g., ICD codes) or described in the baseline questionnaires |  |  |
| **Somatic comorbidity** | Diagnoses in the medical records (e.g., ICD codes) or described in the baseline questionnaires |  |  |
| **Comorbid autoimmune disorder** | Specify which autoimmune disorder (e.g., rheumatoid arthritis) |  |  |
| **Heredity for psychiatric comorbidity** | Specify if 1^st^, 2^nd,^ 3^rd^ (etc…) degree relative and describe the comorbidity (e.g., depression, schizophrenia etc.) |  |  |
| **Heredity for autoimmune disorder** | Specify if 1^st^, 2^nd,^ 3^rd^ (etc…) degree relative and describe the comorbidity (e.g., rheumatoid arthritis) |  |  |
| **Brain injury or head trauma** | If yes, specify |  |  |
| **Smoking** |  |  |  |
| **Educational level** |  | - |  |
| **Employment** |  |  |  |
| **Sickness benefit** |  |  |  |
| **Referral** | E.g., from the neurology department | - |  |
| **Inpatient or outpatient care at the time of the assessment** |  | - |  |
| **AUDIT** | Total score and date |  |  |
| **DUDIT** | Total score and date |  |  |
| **Medication** | At baseline if possible | - |  |
| **Immunomodulatory therapy** | If yes, specify which treatment  E.g., plasmapheresis, corticosteroids, IVIG, Rituximab, NSAIDs, Antibiotics, Antivirals, TNF-inhibitors etc. |  |  |
| **Documented clinical improvement of psychiatric symptoms/function by immunomodulatory treatment** | If yes, specify |  |  |
|  | | | |
| 1. **Brain imaging** | | | |
|  | **Explanation/ examples** | **Data**  **Yes=1**  **No=0** | **Comments/**  **specification** (text) |
| **MRI** | If yes, specify date |  |  |
| **MRI – abnormal changes** |  |  |  |
| **PET** | If yes, specify date |  |  |
| **PET – abnormal changes** |  |  |  |
| **EEG** | If yes, specify date |  |  |
| **EEG – abnormal changes** |  |  |  |
| **EEG – treatment that might affect the results?** | Medication, ECT |  |  |
|  | | | |
| 1. **CSF analysis** | | | |
|  | **Explanation/ examples** | **Cate-gory**  Yes=1  No=0  N/A  Value outside of reference range=1  Normal value=0 | **Exact value** |
| **LP performed** | If yes, specify date |  |  |
| **CSF – WBC** |  |  |  |
| **CSF – Albumin quotient** |  |  |  |
| **CSF – IgG quotient** |  |  |  |
| **CSF – IgG indices** |  |  |  |
| **CSF – Oligoclonal bands** |  |  |  |
| **CSF – Neuronal antibodies** | If positive, specify which |  |  |
| **CSF - Tau** |  |  |  |
| **CSF- GFAP** |  |  |  |
| **CSF - NFL** |  |  |  |
|  | | | |
| 1. **Blood analysis** | | | |
|  | **Explanation/examples** | Value outside of reference range = 1  Normal = 0  N/A | **Exact value** |
| **S- neuronal antibodies** | If positive, specify which |  |  |
| **WBC** |  |  |  |
| **CRP** |  |  |  |
| **Serum protein electrophoresis** |  |  |  |
| **TPO-antibodies** |  |  |  |
|  | | | |
| 1. **Psychiatric rating scales** | | | |
|  |  | **Score** |  |
| **Brief psychiatric rating scale** |  |  |  |
| 1_Somatic concern |  |  |  |
| 2_Anxiety |  |  |  |
| 3_Depression |  |  |  |
| 4_Suicidality |  |  |  |
| 5_Guilt |  |  |  |
| 6_Hostility |  |  |  |
| 7_Elated mood |  |  |  |
| 8_Grandiosity |  |  |  |
| 9_Suspicisousness |  |  |  |
| 10_Hallucinations |  |  |  |
| 11_Unusual thought content |  |  |  |
| 12_Bizarre behaviour |  |  |  |
| 13_Self-neglect |  |  |  |
| 14_Disorientation |  |  |  |
| 15_Conceptual disorganisation |  |  |  |
| 16_Blunted affect |  |  |  |
| 17_Emotional withdrawal |  |  |  |
| 18_Motor retardation |  |  |  |
| 19_Tension |  |  |  |
| 20_Uncooperativeness |  |  |  |
| 21_Excitement |  |  |  |
| 22_Distractibility |  |  |  |
| 23_Motor hyperactivity |  |  |  |
| 24_Mannerisms and posturing |  |  |  |
| Total |  |  |  |
| **Busch-Francis catatonia rating scale** |  |  |  |
| 1_Excitement |  |  |  |
| 2_Immobility/stupor |  |  |  |
| 3_Mutism |  |  |  |
| 4_Staring |  |  |  |
| 5_Posturing/catalepsy |  |  |  |
| 6_Grimacing |  |  |  |
| 7_Echopraxia/echolalia |  |  |  |
| 8_Steroptypy |  |  |  |
| 9_Mannerism |  |  |  |
| 10_Verbigeration |  |  |  |
| 11_Rigidity |  |  |  |
| 12_Negativism |  |  |  |
| 13_Waxy_flexibility |  |  |  |
| 14_Withdrawal |  |  |  |
| 15_Impulsivity |  |  |  |
| 16_Automatic_obedience |  |  |  |
| 17_Mitgehen |  |  |  |
| 18_Gegenhalten |  |  |  |
| 19_Ambitendency |  |  |  |
| 20_Grasp_reflex |  |  |  |
| 21_Perseveration |  |  |  |
| 22_Combativeness |  |  |  |
| 23_Autonomic_abnormality |  |  |  |
| Total |  |  |  |
| **CGI** |  |  |  |

**CSF routine analysis reference values**

- IgG indices (ref < 0.63).
- Age-related CSF/plasma albumin quotient (ref: age > 6 months to 45 years, < 6.8; age ≥ 45 years, < 10.2)
- White blood cell count (ref < 5 cells per 106/L).

**CNS damage biomarkers reference values**

| **NfL** | | **GFAP** | |
| --- | --- | --- | --- |
| **Age (in years)** | **Reference value (ng/L)** | **Age (in years)** | **Reference value (ng/L)** |
| <30 | <380 | <20 | <175 |
| 30-39 | <560 | 20-59 | <750 |
| 40-59 | <890 | ≥60 | <1,250 |
| ≥60 | <1,850 |  | |

| **Total Tau** | |
| --- | --- |
| **Age (in years)** | **Reference value (ng/L)** |
| <18 | <250 |
| 18-44 | <300 |
| ≥45 | <400 |
