# Supplementary material for: An exploratory study of the damage markers NfL, GFAP, and t-Tau, in cerebrospinal fluid and other findings from a patient cohort enriched for suspected autoimmune psychiatric disease
Source: Transl Psychiatry. 2024 Jul 24;14:304. doi: 10.1038/s41398-024-03021-8 (PMC11269634; doi:10.1038/s41398-024-03021-8)
Supplement: Supplementary file 2 — Supplementary Tables 1–7 and Supplementary Figure legends [file 41398_2024_3021_MOESM2_ESM.docx]

| **Supplementary table 1A:** CNS damage biomarkers relationship with clinical red flags | | | | | | | | | | | | | | | |
| --- | --- | --- | --- | --- | --- | --- | --- | --- | --- | --- | --- | --- | --- | --- | --- |
|  | Atypical presentation | | | Rapid onset | | | Prodromal infection | | | Comorbid autoimmune disorder | | | Comorbid tumours | | |
|  | Yes | No | p | Yes | No | p | Yes | No | p | Yes | No | p | Yes | No | p |
| NfL above reference, n/N (%) | 11/94 (12) | 3/30 (10) | 1.000 | 6/66  (9) | 8/58 (14) | 0.409 | **2/48**  **(4)** | **12/76**  **(16)** | **0.046*** | **9/43**  **(21)** | **5/81**  **(6)** | **0.018*** | 1/8  (13) | 13/116  (11) | 1.000 |
| t-Tau above reference, n/N (%) | 13/90  (14) | 3/30  (10) | 0.758 | 6/65  (9) | 10/55  (18) | 0.151 | 5/47  (11) | 11/73  (15) | 0.486 | 7/41  (17) | 9/79  (11) | 0.385 | 2/8  (25) | 14/98  (14) | 0.289 |
| GFAP above reference, n/N (%) | **14/94 (15)** | **0/30**  **(0)** | **0.021*** | **11/66**  **(17)** | **3/55**  **(5)** | **0.044*** | **10/48**  **(21)** | **4/72**  **(6)** | **0.008**** | 2/43  (5) | 12/81  (15) | 0.135 | 0/8  (0) | 14/116  (12) | 0.595 |

**Supplementary Tables - Syk and Tornvind et al**

| **Supplementary table 1A:** CNS damage biomarkers relationship with clinical red flags | | | | | | | | | | | | | | | |
| --- | --- | --- | --- | --- | --- | --- | --- | --- | --- | --- | --- | --- | --- | --- | --- |
|  | Suspected malignant neuroleptic syndrome ^a^ | | | Any abnormal neurological findings | | | Motor symptoms | | | Novel seizures ^a^ | | | Catatonia | | |
|  | Yes | No | p | Yes | No | p | Yes | No | p | Yes | No | p | Yes | No | p |
| NfL above reference, n/N (%) | ½  (50) | 13/122  (11) | 0.214 | 5/48  (10) | 9/76  (12) | 0.807 | 1/18  (6) | 13/106  (12) | 0.690 | 0/3  (0) | 14/121  (12) | 1.000 | 3/31  (10) | 11/93  (12) | 1.000 |
| t-Tau above reference, n/N (%) | 0/2  (0) | 16/118  (14) | 1.000 | 8/46  (17) | 8/74  (11) | 0.303 | 3/17  (18) | 13/103  (13) | 0.699 | 0/3  (0) | 16/117  (14) | 1.000 | **8/30**  **(27)** | **8/90**  **(9)** | **0.026*** |
| GFAP above reference, n/N (%) | 0/2  (0) | 14/122  (12) | 1.000 | 5/48  (10) | 9/76  (12) | 0.807 | 3/18  (17) | 11/106  (10) | 0.427 | 0/3  (0) | 14/121  (12) | 1.000 | 6/31  (19) | 8/93  (9) | 0.112 |

^a^ The very low prevalence of suspected malignant neuroleptic syndrome or novel seizures makes the statistical testing unreliable for these variables

Y = Yes; N = No

| **Supplementary table 1B:** CNS damage biomarkers relationship with psychiatric symptomatology | | | | | | | | | | | | | | | |
| --- | --- | --- | --- | --- | --- | --- | --- | --- | --- | --- | --- | --- | --- | --- | --- |
|  | Psychosis | | | Cognitive symptoms | | | Obsessions-compulsions | | | Tics | | | Psychomotor retardation | | |
|  | Yes | No | p | Yes | No | p | Yes | No | p | Yes | No | p | Yes | No | p |
| NfL above reference, n/N (%) | 9/73  (12) | 5/51  (10) | 0.662 | 9/74  (12) | 5/50  (10) | 0.709 | **2/53**  **(4)** | **12/71**  **(17)** | **0.022*** | 1/8  (13) | 13/115  (11) | 1.000 | 3/20  (15) | 11/104  (11) | 0.698 |
| t-Tau above reference, n/N (%) | 7/69  (10) | 9/51  (18) | 0.232 | 11/72  (15) | 5/48  (10) | 0.443 | 7/50  (14) | 9/70  (13) | 0.856 | 2/8  (25) | 14/111  (13) | 0.293 | 0/20  (0) | 16/100  (16) | 0.071 |
| GFAP above reference, n/N (%) | 6/73  (8) | 8/51  (16) | 0.196 | 5/74  (7) | 9/50  (18) | 0.052 | 8/53  (15) | 6/71  (8) | 0.248 | **4/8**  **(50)** | **10/115**  **(9)** | **0.006**** | 3/20  (15) | 11/104  (11) | 0.698 |

| **Supplementary table 1B:** CNS damage biomarkers relationship with psychiatric symptomatology | | | | | | | | | | | | | | | |
| --- | --- | --- | --- | --- | --- | --- | --- | --- | --- | --- | --- | --- | --- | --- | --- |
|  | Observed affective dysregulation symptoms | | | Mania | | | Agitation/aggression | | | Hypersomnia | | | Insomnia | | |
|  | Yes | No | p | Yes | No | p | Yes | No | p | Yes | No | p | Yes | No | p |
| NfL above reference, n/N (%) | **12/65**  **(19)** | **2/59**  **(3)** | **0.008**** | 2/15  (13) | 11/108  (10) | 0.659 | 2/26  (8) | 12/98  (12) | 0.732 | 0/11  (0) | 14/99  (14) | 0.612 | 5/44  (11) | 9/80  (11) | 1.000 |
| t-Tau above reference, n/N (%) | 8/65  (8) | 8/55  (15) | 0.719 | 2/13  (15) | 14/106  (13) | 0.687 | 3/26  (12) | 13/94  (14) | 1.000 | 0/11  (0) | 16/109  (15) | 0.356 | 7/40  (18) | 9/80  (11) | 0.342 |
| GFAP above reference, n/N (%) | 7/65  (11) | 7/59  (12) | 0.847 | 1/15  (7) | 13/108  (12) | 1.000 | 1/26  (4) | 13/98  (13) | 0.297 | 1/11  (9) | 13/113  (12) | 1.000 | 4/44  (9) | 10/80  (13) | 0.768 |

| **Supplementary table 1C:** Comparison of BPRS and BFCRS total score and factor scores between patients with and without CNS damage biomarkers (NfL, Tau, GFAP) (p-values from the Mann-Whitney tests) | | | | | | | | | | |
| --- | --- | --- | --- | --- | --- | --- | --- | --- | --- | --- |
|  | BPRS Total score | BPRS F1  Depressed/anxiety | BPRS F2  Psychosis | BPRS F3  Negative symptoms/  retardation | BPRS F4  Activation | BFCRS Total score | BFCRS F1  Negative/withdrawal | BFCRS F2  Automatic | BFCRS F3  Repetitive/echo | BFCRS F4  Agitated/resistive |
| NfL above reference | 0.205 | 0.364 | 0.188 | 0.834 | 0.529 | 0.960 | 0.205 | 0.303 | 0.837 | 0.754 |
| t-Tau above reference | 0.876 | 0.512 | 0.611 | 0.492 | **0.033*** | 0.097 | 0.516 | 0.769 | 0.053 | **0.041*** |
| GFAP above reference | **0.033*** | **0.021*** | 0.483 | 0.457 | 0.243 | 0.205 | 0.121 | 0.211 | 0.106 | 0.080 |

| **Supplementary table 1D:** CNS damage biomarkers relationship with MRI and EEG findings | | | | | | | | | | | | |
| --- | --- | --- | --- | --- | --- | --- | --- | --- | --- | --- | --- | --- |
|  | MRI Any findings | | | MRI WMC | | | MRI Atrophy | | | EEG background changes | | |
|  | Y | N | p | Y | N | p | Y | N | p | Y | N | p |
| NfL above reference, n/N (%) | 8/57  (14) | 3/29 | 0.743 | 7/36  (19) | 4/50  (8) | 0.189 | 6/43  (14) | 5/43  (12) | 0.747 | **6/19**  **(32)** | **4/48**  **(8)** | **0.025*** |
| t-Tau above reference, n/N (%) | 9/53  (17) | 4/29  (14) | 1.000 | 8/34  (24) | 5/48  (10) | 0.109 | 6/40  (15) | 7/42  (17) | 0.836 | 4/19  (21) | 4/46  (9) | 0.218 |
| GFAP above reference, n/N (%) | 9/57  (16) | 5/29  (17) | 1.000 | 4/36  (11) | 10/50  (20) | 0.271 | 7/43  (16) | 7/43  (16) | 1.000 | 3/19  (16) | 7/48  (16) | 1.000 |
| WMC = White matter changes | | | | | | | | | | | | |

**Supplementary table 2: The distribution of MRI white matter changes (WMC) in relation to age categories**

| Age group | MRI available (n=88) | N with ”any WMC” |
| --- | --- | --- |
| <30 | 48 | 9 (19%) |
| 30-39 | 18 | 13 (72%) |
| 40-49 | 9 | 4 (44%) |
| 50-59 | 7 | 5 (71%) |
| 60-69 | 5 | 4 (80%) |
| >70 | 1 | 1 (100%) |

| **Supplementary table 3:** Description of the clinical red flags | |
| --- | --- |
| **Clinical red flag** | **Description** |
| Atypical presentation | A broad category including a global impression of atypical trajectory or symptomatology such as disproportionate cognitive deficits or hypersomnia |
| Rapid onset | (Sub)acute onset of severe psychiatric symptoms defined as a rapid progression of <3 months |
| Prodromal infection | A temporal association of onset/deterioration of psychiatric/neurological symptoms with suspected or confirmed infection |
| Comorbid autoimmune disorder | For example, systemic lupus erythematosus |
| Comorbid tumours | Malignant or benign |
| Suspected malignant neuroleptic syndrome | Noted in the medical records |
| Any abnormal neurological findings | Any abnormal findings in the neurological examination |
| Motor symptoms | For example, involuntary movements or dystonia |
| New-onset seizures | Novel seizures in conjunction with or after disease onset |
| Catatonia | Catatonia symptoms reported by the assessing or treating clinician in the medical records or a total Busch Francis Catatonia Rating Scale score of >3 points |

| **Supplementary table 4:** Fulfilment of previously suggested diagnostic criteria for autoimmune psychiatric disorders | | | | |
| --- | --- | --- | --- | --- |
|  | **Autoimmune psychosis^a^**  **(N=74)** | **Autoimmune OCD^b^**  **(N=53)** | **Autoimmune other psychiatry^c^**  **(N=25)** | **Total^d^**  **(N=127)** |
| Possible, n (%) | 29 (40%) | 27 (51%) | 11 (44%) | 60 (47%) |
| Probable, n (%) | 2 (3%) | 4 (8%) | 1 (4%) | 6 (5%) |
| Definite,  n (%) | 1 (1%) | 1 (2%) | 1 (4%) | 3 (2%) |
| ^a^ Pollak et al 2020 criteria, ^b^ Endres et al 2022 criteria, ^c^ Based on a modification of the Endres et al 2022 criteria in cases with other psychiatric manifestations, ^d^ Autoimmune psychosis, autoimmune OCD and/or autoimmune other psychiatry | | | | |

| **Supplementary table 5:** Description of the three patients with positive anti-neuronal IgG antibody findings against the NMDAR in CSF | | | | | | | | | | | |
| --- | --- | --- | --- | --- | --- | --- | --- | --- | --- | --- | --- |
| **Anti-neuronal antibody** | **Sex**  **Age** | **Onset to testing (years)** | **Clinical red flags** | **Primary symptoms** | **CSF basic findings** | **CNS damage markers** | **Other findings** | **MRI** | **EEG** | **FDG-PET** | **Immuno-modulatory treatment** |
| Serum and CSF IgG NMDAR abs | F  30s | >20 | Rapid onset, prodromal infection, known autoimmune disorder (antiphos-pholipid syndrome) | Obsessions-compulsionsCognitive dysfunction. Fatigue. Depression. Insomnia. | Negative | Normal | Elevated fibrinogenDecreased serum albumin. Elevated CRP (20 mg/L). | NA | NA. | NA | - |
| Serum and CSF IgG NMDAR abs | F  30s | <1 | Atypical presentation, rapid onset | Personality change. Hypomania. Cognitive dysfunction. Fatigue. Visual impairment. ParaesthesiaAphasia. Ataxia. Subjective weakness in right arm. | Pleocytosisunmatched OCBs  Elevated IgG-index | Elevated NfL | - | Multiple white matter lesions in the brain and spinal cord consistent with demyelinating disease. Several with contrast enhancement. | Diffuse slowing, focal slowing left frontotemporal lobe and slow posterior dominant rhythm. No epileptiform activity. | NA | Distinct improve-ment with steroids. Later some further improve-ment  with rituximab. |
| CSF IgG NMDAR abs | F  60s | <1 | Atypical presentation  Rapid onset  Known autoimmune disorder (rheumatoid arthritis) | Seizures. Psychosis. Aggression. Depression. Severe anxiety. Left side facial twitches. | OCBs  Elevated IgG index | Normal | Elevated fibrinogen in serum. Elevated CRP (6 mg/L) | Extensive focal white matter lesions, disseminated foci with restricted diffusion. Bleeding residues and leptomeningeal contrast enhancement. | Normal | Asymmetrical slightly hypermeta-bolic changes in the lateral frontal lobes^1^ and right parietal lobe including frontal areas associated with anxiety or fear^1^ and sensory-motor areas for the left arm and left side of the face. | Improve-ment after treatment with rituximab. |
| ^1^ Increased FDG uptake or hypermetabolism in the frontal lobes may be associated with positive psychiatric symptoms (Shinto, A.S. 2014, Soyka, M. 2005) whereas cerebellar hypometabolism is more unspecific but could be a side effect of drug treatment (Seitz, R.J. 1996, Bairamian, D. 1986). Abs=Antibodies; CNS=Central nervous system; CSF=Cerebrospinal fluid; EEG=Electroencephalogram; FDG-PET = 2-deoxy-2[F-18]fluoro-D-glucose positron emission tomography; MRI=Magnetic Resonance Imaging; NA=Not assessed; OCBs=Oligoclonal bands; | | | | | | | | | | | |

| **Supplementary table 6:**  Description of the five patients with positive anti-neuronal IgG antibody findings against intracellular antigen targets in serum | | | | | | | | | | | |
| --- | --- | --- | --- | --- | --- | --- | --- | --- | --- | --- | --- |
| **Anti-neuronal antibody** | **Sex, age** | **Time between onset and testing (years)** | **Clinical red flags** | **Primary symptoms** | **CSF basic findings** | **CNS damage biomarkers** | **Other findings** | **MRI** | **EEG** | **FDG-PET** | **Immunomodulatory treatment** |
| Anti-Ma2/Ta IgG abs | F  20s | <2 | Rapid onset | Obsessions-compulsions. Depression. Anxiety. Suicidality. Recurrent infections. Hyperreflexia. | Negative | Normal | Elevated fibrinogen in serum. | Mild cortical atrophy | NA | Hypometabolic changes in the cerebellum^1^. | Slight temporary improvement with steroids |
| Anti-Ma2/Ta IgG abs | F  20s | <7 | Atypical presentation  Known autoimmune thyroid disorder | Obsessions. Cognitive dysfunction. Psychomotor retardation. Depression. Anxiety. Eating disorder. Paraesthesia. | Negative | Normal | Anti-TPO Abs: Positive. | Normal | Normal | Hypometabolic changes in the cerebellum, vermis, brain stem and deep central structures. | - |
| Anti-GAD65 IgG abs | M  40s | <10 | Atypical presentation  Known autoimmune disorder (diabetes type 1) | Psychosis. Atypical obsessions. | Negative | Normal | Elevated fibrinogen in serum. Borrelia IgM: Positive. Borrelia IgG: Negative. | NA | NA | NA | - |
| Anti-Zic4 IgG abs | M  20s | <2 | Atypical presentation  Rapid onset | Psychosis. Obsessions. Insomnia. Anxiety. Light- and sound sensitivity. | Negative | Normal | No | Mild cortical atrophy | Normal | Normal | - |
| Anti-Zic4 IgG abs | M  40s | <5 | Atypical presentation | Psychosis (delusions). Headache. Attacks with shivers and piloerection. Anxiety. | Elevated AQ | Normal | Borrelia IgM: Positive. Borrelia IgG: Negative | Mild cortical atrophy | Normal | Slightly hypometabolic changes in the anterior cingulate, brainstem and cerebellum. | **-** |
| ^1^ Increased FDG uptake or hypermetabolism in the frontal lobes may be associated with positive psychiatric symptoms (Shinto, A.S. 2014, Soyka, M. 2005), whereas cerebellar hypometabolism is more unspecific but could be a side effect of drug treatment (Seitz, R.J. 1996, Bairamian, D. 1986). Abs=Antibodies; AQ=Albumin quotient; CNS=Central nervous system; EEG=Electroencephalogram; FDG-PET = 2-deoxy-2[F-18]fluoro-D-glucose positron emission tomography; MRI=Magnetic Resonance Imaging; NA=Not assessed. | | | | | | | | | | | |

| **Supplementary table 7:** Distribution of the CSF variables in the population | | | | | | | |
| --- | --- | --- | --- | --- | --- | --- | --- |
|  | CSF NfL | CFS t-Tau | CSF GFAp | CSF IgG ratio | CSF IgG indices | CSF albumin quotient | CSF white blood cell count (poly+mono) |
| N | 124 | 120 | 124 | 122 | 122 | 122 | 125 |
| Median | 260 | 207 | 300 | 2.22 | 0.46 | 4.75 | 1 |
| 25^th^ percentile | 190 | 152 | 193 | 1.65 | 0.43 | 3.50 | 0 |
| 75^th^ percentile | 490 | 278 | 380 | 3.30 | 0.50 | 6.43 | 2 |
| Minimum | 49 | 74 | 69 | 0.86 | 0.30 | 1.90 | 0 |
| Maximum | 8290 | 1400 | 1150 | 6.86 | 1.85 | 13.90 | 21 |

**Supplementary Figure 1.** Flow-chart of the inclusion process

**Supplementary Figure 2.** Bubble plots illustrating the importance of different clinical variables for the prediction of elevated CNS damage biomarkers (A: GFAP, B: NfL, C: t-Tau)

The x-axis shows the prevalence of the variable in patients with elevated GFAP, NfL or t-Tau. The higher the x-value, the higher the prevalence. The y-axis shows how important a variable is for the accuracy of the models. A higher y-value suggests higher importance of the variable for the correct prediction of elevated GFAP, NfL or t-Tau. The size of the bubbles demonstrates the prevalence of the variable in the total patient population. The larger the bubble, the more common the variable is in the total population. The colour of the bubbles shows the odds ratio (OR) of the variable, comparing patients with and without elevated GFAP, NfL or t-Tau. OR <1 indicates that the variable is more common in patients with normal GFAP, NfL or t-Tau. OR=1 means there is no difference between patients with and without elevated GFAP, NfL or t-Tau. OR >1 means the variable is more common in patients with elevated GFAP, NfL or t-Tau.
