# Supplementary figures and images for: An exploratory study of the damage markers NfL, GFAP, and t-Tau, in cerebrospinal fluid and other findings from a patient cohort enriched for suspected autoimmune psychiatric disease
Source: Transl Psychiatry. 2024 Jul 24;14:304. doi: 10.1038/s41398-024-03021-8 (PMC11269634; doi:10.1038/s41398-024-03021-8)

**Figure 1. Flow diagram of the inclusion process**

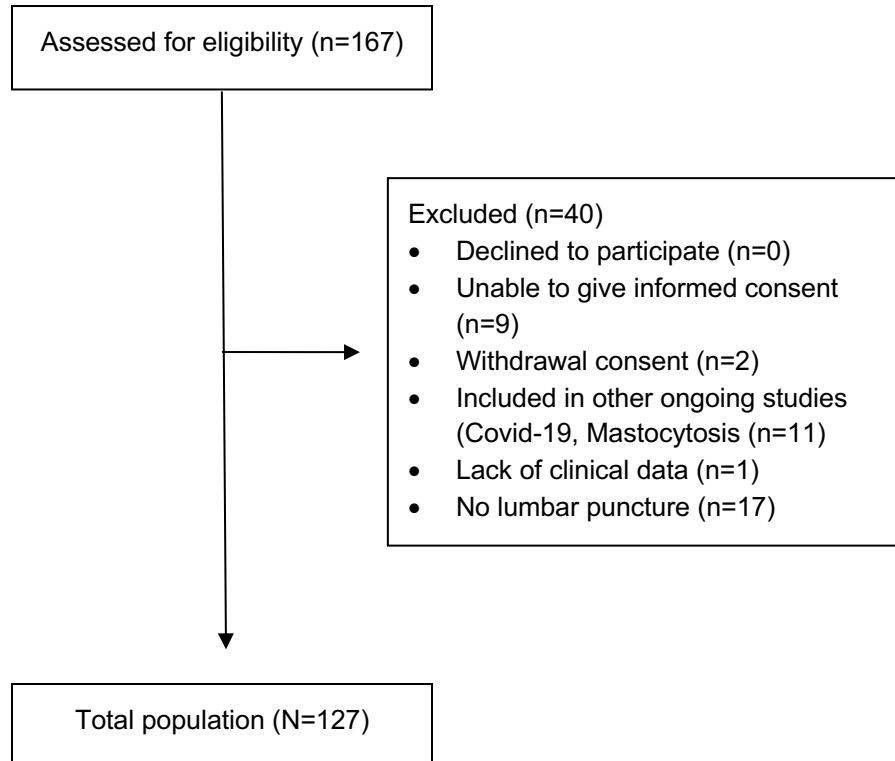

Supplement: Supplementary file 3 — Supplementary Figure 1. Flow-chart of the inclusion process [file 41398_2024_3021_MOESM3_ESM.pdf]

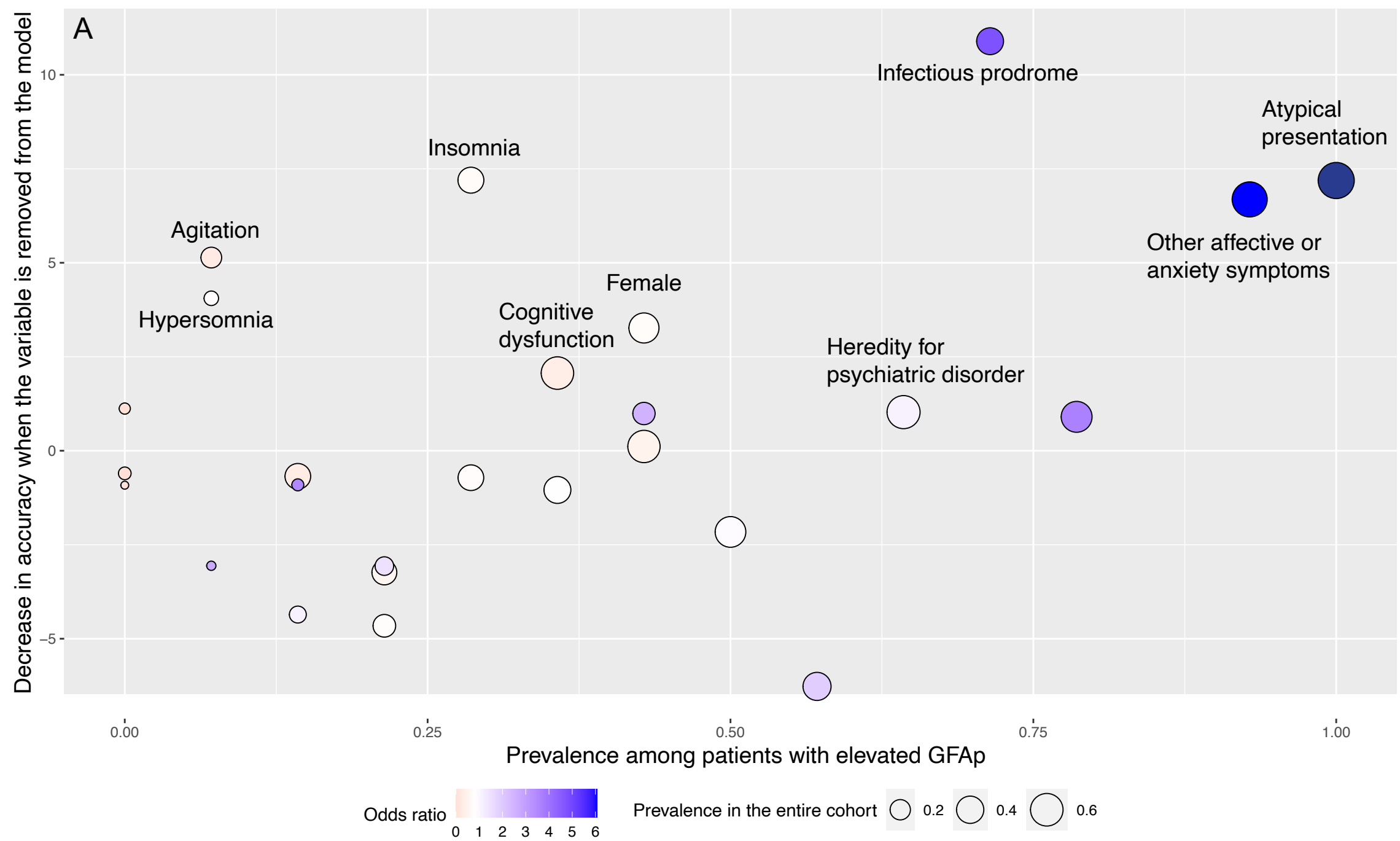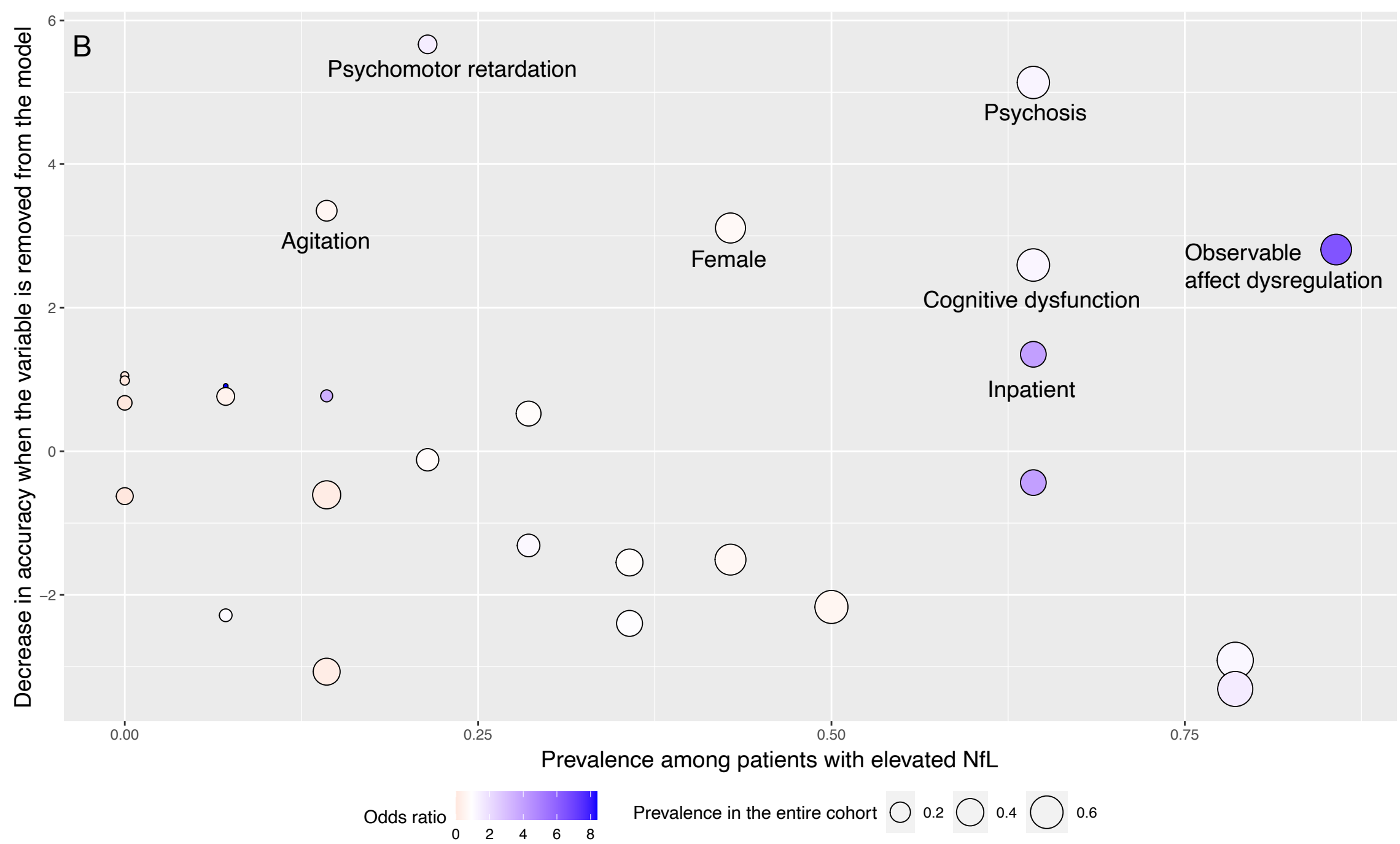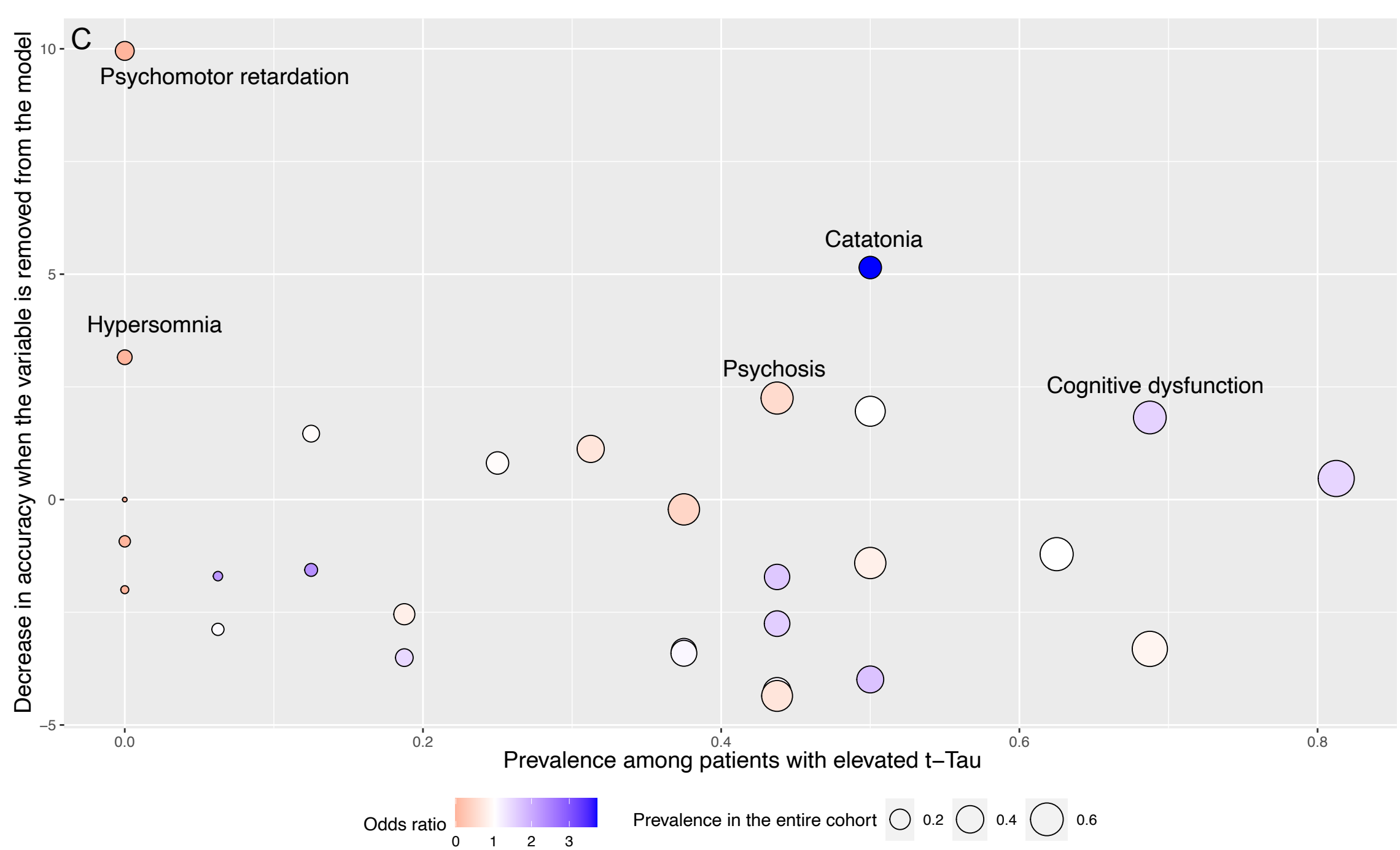

Supplement: Supplementary file 4 — Supplementary Figure 2.Effect size for the predictive importance of the explanatory variables [file 41398_2024_3021_MOESM4_ESM.pdf]
